# Effect of Post-Activation Performance Enhancement in Combat Sports: A Systematic Review and Meta-Analysis-Part II: Specific Performance Indicators

**DOI:** 10.3390/jfmk11020157

**Published:** 2026-04-16

**Authors:** Artur Terbalyan, Karol Skotniczny, Marcin Żak, Jakub Jarosz, Robert Roczniok

**Affiliations:** Institute of Sport Sciences, Jerzy Kukuczka Academy of Physical Education, 40-065 Katowice, Poland; k.skotniczny@awf.katowice.pl (K.S.); m.zak@awf.katowice.pl (M.Ż.); j.jarosz@awf.katowice.pl (J.J.)

**Keywords:** strength and conditioning, martial arts, potentiation, taekwondo, muscle strength

## Abstract

**Objectives**: Post-activation performance enhancement (PAPE) has been explored for its potential to improve performance in combat sports. This part II of the systematic review and meta-analysis investigated the acute effects of PAPE protocols on sport-specific performance outcomes and evaluated the influence of moderating variables, specifically competitive level and training experience. **Methods**: A PRISMA-guided search (2010–2024) identified 13 studies examining PAPE in combat sports athletes. Inclusion criteria required human trials using defined PAPE protocols and evaluating sport-specific tests, primarily the Frequency Speed of Kick Test (FSKT-10) and the Taekwondo-Specific Agility Test (TSAT). A random-effects meta-analysis (Hedges’ g) was conducted on data from 176 athletes. **Results**: The meta-analysis revealed a profound moderating effect of training status on PAPE responsiveness. For the FSKT-10, amateur athletes demonstrated large, significant improvements (g = 1.28, *p* < 0.001), whereas elite athletes showed trivial, non-significant changes (g = 0.11, *p* = 0.357). Similarly, athletes with <6 years of training experience exhibited substantially larger enhancements in both FSKT-10 (g = 1.60) and TSAT agility (g = −1.64) compared to their more experienced (>6 years) counterparts (g = 0.42 and g = −0.65, respectively). Furthermore, dynamic and biomechanically specific conditioning activities (e.g., repeated high-intensity techniques) were most effective at driving sport-specific potentiation. **Conclusions**: PAPE protocols may enhance acute sport-specific performance when utilizing dynamic, highly specific conditioning activities. However, a possible “ceiling effect” may blunt this potentiation in elite and highly experienced athletes, suggesting a potential need for highly individualized priming strategies at the top competitive levels, specifically in taekwondo.

## 1. Introduction

The physical demands of combat sports are characterized by the multifaceted interaction between neuromuscular prerequisites and technical execution under intermittent, high-intensity conditions [1,2]. Although fundamental qualities—specifically strength, power, and speed—constitute the physiological foundation, competitive efficacy is contingent upon the efficient translation of these capacities into discipline-specific movement patterns within the sport’s kinematic and kinetic constraints [3].

In Part I of this work [4], we meta-analyzed the post-activation performance enhancement (PAPE) on general neuromuscular indices (e.g., ballistic jumps, linear sprints, or jumping ability), which are characterized by increased force production, rate of force development, and power output—phenomena that occur following a high-intensity conditioning activity and have gained considerable attention as a practical pre-competition priming strategy in combat sports. The pooled findings revealed a small overall effect (ES = 0.136) that did not differ significantly between amateur and elite athletes, suggesting that PAPE exerts a modest and relatively uniform influence on general ballistic performance regardless of competitive level. However, such general neuromuscular tests may lack ecological validity for competition-relevant performance, which is inherently governed by complex motor control and open-skill technical execution [3]. Consequently, verifying the translational utility of acute priming strategies necessitates determining whether the conditioning activity (CA) effectively potentiates these specific motor tasks in discipline-relevant contexts, rather than extrapolating findings from isolated athletic tasks.

To address these ecological limitations, Part II systematically examines sport-specific performance outcomes, prioritizing discipline-relevant field tests and task simulations. Depending on the specific combat discipline, assessment protocols encompass high-frequency striking/kicking tasks, specialized agility maneuvers, or grappling-based efforts. For instance, Taekwondo research frequently utilizes the Frequency Speed of Kick Test (FSKT) and the Taekwondo-Specific Agility Test (TSAT), both of which have demonstrated robust psychometric properties (i.e., reliability and construct validity) within this population [5,6]. Analogous sport-specific testing frameworks have been validated in other grappling disciplines, such as the Special Judo Fitness Test (SJFT) in judo [7].

Accordingly, the principal objective of this second part was to systematically review and meta-analyze the acute effects of PAPE on sport-specific performance outcomes in combat sports athletes. Secondarily, the study aimed to elucidate the influence of moderating variables—specifically competitive level, discipline and training experience—on the observed heterogeneity in responsiveness.

## 2. Materials and Methods

### 2.1. Search Strategy

This systematic review was conducted in accordance with the Preferred Reporting Items for Systematic Reviews and Meta-Analyses (PRISMA) guidelines [8,9]. Full PRISMA checklist can be found in Supplementary File S1. The protocol for this systematic review was retrospectively registered at Open Science Framework (osf.io/ja84d). The data collection process involved four distinct phases: literature verification, screening, eligibility assessment, and final selection [10]. A comprehensive search of electronic databases—including PubMed/Medline, SPORTDiscus, Web of Science, Scopus, EBSCO, and Google Scholar—was performed by the research team to identify relevant studies published between January 2010 and March 2024. The search strategy employed Boolean operators to combine terms related to combat sports (e.g., ‘combat sport*’, ‘MMA’, ‘judo’, ‘wrestl*’) with terminology specific to conditioning activities and potentiation phenomena (‘post-warm-up’, ‘post-activation potentiation’, ‘post-activation performance enhancement’). Truncation symbols (‘*’) were utilized to capture all grammatical variations. Additionally, the reference lists of the included articles and relevant recent systematic reviews were manually screened (backward snowballing) to identify any further eligible studies that might have been omitted during the electronic database search. The protocol for this systematic review was retrospectively registered at the Open Science Framework (osf.io/ja84d).

### 2.2. Inclusion and Exclusion Criteria

This review was restricted to original, peer-reviewed research articles published in English with full-text availability; review papers, theses, dissertations, and unpublished abstracts were excluded. Eligibility was limited to experimental trials involving healthy human subjects actively engaged in recognized combat sports disciplines (e.g., boxing, judo, karate, taekwondo, Brazilian jiu-jitsu (BJJ), mixed martial arts (MMA), wrestling, kickboxing, or Muay Thai). Participants were required to be free of musculoskeletal injuries or health conditions capable of impairing neuromuscular function. Furthermore, eligible studies had to implement distinct post-activation potentiation (PAP) or PAPE protocols, compare them against valid control conditions (such as passive rest or standard warm-up), and report quantitative performance outcomes. The selection criteria were standardized using the PICOS framework [9] in alignment with PRISMA guidelines (Table 1).

### 2.3. Text Screening

The screening process was conducted independently by two investigators (AT and KS). Initial selection involved the assessment of titles and abstracts based on the a priori eligibility criteria. Subsequently, the full texts of potentially relevant articles were reviewed by the same researchers to confirm final inclusion. Any discrepancies between the reviewers were resolved through discussion until a consensus was achieved.

### 2.4. Data Extraction, Study Coding

The extracted information included the study authors, year of publication, study design, age, sex, competitive level, the combat sports discipline, CAs with training variables, and outcomes. In addition to the primary study characteristics, the following moderating variables were extracted and coded for subsequent subgroup analyses: (1) competitive level, classified as amateur or elite; (2) years of training experience, dichotomized as less than six years or more than six years based on the distribution of the available data; and (3) conditioning activity type, classified as sport-specific (e.g., repeated high-intensity kicking sequences), plyometric, or traditional resistance exercise. The first and second authors gathered the data through the blind method. The authors collected the means with standard deviations (SD) of pre- and post-conditioning performance test results. The third author was responsible for the initial screening and selection process. Any missing data were obtained by direct request to the corresponding author. In the absence of feedback, ImageJ© software (ImageJ v. 1.54d, National Institute of Health, Bethesda, MD, USA) was employed to obtain the requisite data. In cases where data were unavailable, the study was excluded. Effect sizes were calculated from the means and standard deviations of the pre-and post-intervention data. The results of the random-effects meta-analysis are presented in a forest plot. The minimum number of studies included in the meta-analysis was five. All studies meeting the inclusion criteria were carefully reviewed to document relevant study characteristics and were tabulated in an Excel spreadsheet (Microsoft Corporation, Redmond, WA, USA).

### 2.5. Quality Assessment

Methodological quality and risk of bias were assessed independently by two reviewers (AT and KS) using the Physiotherapy Evidence Database (PEDro) scale [10]. Each item on the scale was scored as 1 (criterion met) or 0 (criterion not met or clearly unspecified). Any discrepancies between the reviewers were resolved through a consensus meeting. Study quality was categorized based on the total score: excellent (9–10), good (6–8), fair (4–5), or poor (0–3). To ensure the robustness of the results, distinct quality thresholds were applied: studies scoring >4 were eligible for the systematic review, whereas a stricter threshold of ≥6 (indicating ‘good’ quality) was required for inclusion in the meta-analysis [11]. To complement the PEDro assessment and address its primary limitation—namely, its development for randomized controlled trials—the certainty of evidence for the primary outcomes (FSKT-10 and TSAT) was additionally evaluated using the Grading of Recommendations Assessment, Development and Evaluation (GRADE) framework.

### 2.6. Meta-Analysis

Meta-analytic procedures were performed using means, standard deviations, and Hedges’ g effect sizes (ES) alongside their 95% confidence intervals (CI), calculated according to the methods of Hedges and Olkin [12]. Hedges’ g was selected to correct for small-sample bias, which was prevalent in the included studies. The magnitude of the effect sizes was interpreted according to the thresholds proposed by Hopkins et al. [13]: trivial (<0.20), small (0.20–0.59), moderate (0.60–1.19), large (1.20–1.99), and very large (≥2.0). Heterogeneity was assessed visually via forest plots and quantified using the I^2^ statistic, where values ≥ 50% indicate substantial heterogeneity. Consequently, a conservative random-effects model was applied to all analyses. Statistical computations were executed using PQStat Software (v. 1.8.2.208; Poznań, Poland), while data visualization was generated with GraphPad Prism 10 (v. 10.1.1., GraphPad Software; San Diego, CA, USA). Due to the limited number of eligible studies, publication bias was not assessed via funnel plots or formal tests, as such methods are prone to misinterpretation with small datasets [14]. Subgroup analyses were conducted using Cochran’s Q test (between-group Q) to evaluate ES differences across categorical moderators (e.g., competitive level and years of practice), with statistical significance defined as *p* < 0.05. For repeated-measures crossover designs where the pre-post or inter-condition correlation coefficients were not reported, a conservative correlation coefficient of r = 0.7 was assumed for the calculation of standard errors. This assumption is justified by the high test-retest reliability typically observed in explosive and combat-specific performance tests, such as vertical jumping tasks [15]. To evaluate the robustness of pooled estimates in subgroups exhibiting substantial heterogeneity, a leave-one-study-out sensitivity analysis was performed. This approach was preferred over leave-one-arm-out analysis given the multi-arm structure of the included studies, in which a single study may contribute several conditioning activity arms derived from the same participant sample. Sequentially removing all arms from a given study provides a more conservative and methodologically appropriate assessment of each study’s influence on the pooled estimate and heterogeneity index. Additionally, to address the potential violation of the statistical independence assumption arising from the inclusion of multiple conditioning activity arms from the same study, a supplementary study-level sensitivity analysis was conducted. For each study contributing more than one conditioning activity arm, individual arm-level effect sizes were aggregated into a single composite estimate using inverse-variance weighting prior to meta-analytic pooling. The resulting study-level dataset was subsequently re-analyzed using the same random-effects model as the primary analysis.

## 3. Results

### 3.1. Literature Search

**Figure 1:**
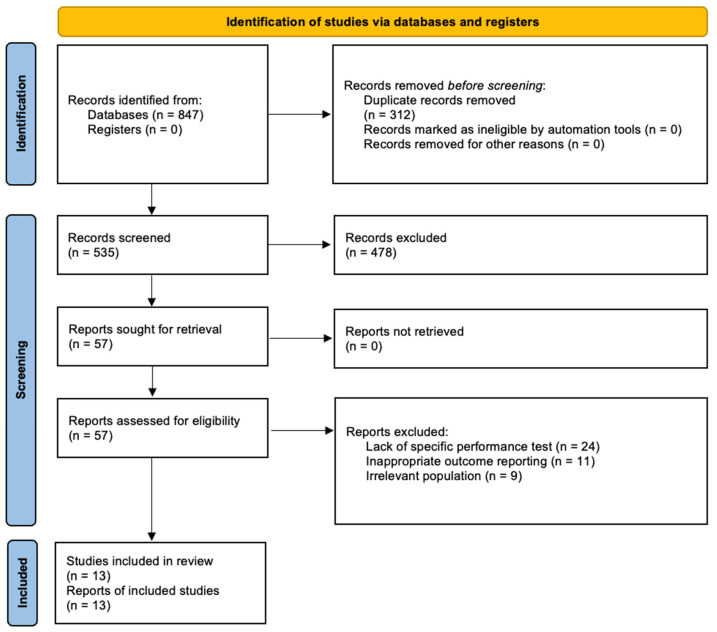
PRISMA diagram of the search and study inclusion process. Databases searched: PubMed/Medline, SPORTDiscus, Web of Science, Scopus, EBSCO, and Google Scholar.

Figure 1 illustrates the screening process, which was organized into three consecutive phases according to the PRISMA guidelines: (i) identification, (ii) screening, and (iii) inclusion in the review. A total of 13 articles were ultimately selected to investigate the PAPE on specific performance tests (SPT) in combat sports.

### 3.2. Quality Assessment

The methodological quality of the included studies was evaluated using the PEDro scale. The mean PEDro score across all 13 studies was 7.8 out of a maximum of 10 points. Based on the established classification criteria, six studies (46%) were rated as excellent (scoring 9–10 points), and seven studies (54%) were classified as good (scoring 6–8 points). None of the included studies were classified as fair or poor. The most commonly unmet criteria across the reviewed literature were the blinding of therapists (Item 6), blinding of subjects (Item 5), and concealed allocation (Item 3). The complete results of the quality assessment are presented in Table 2 below.

### 3.3. Systematic Review

Among included studies, the majority focused on taekwondo (8 articles) [17,18,19,20,21,26,28], followed by boxing (2 articles) [22,23], judo (2 articles) [24,27], and karate (1 article) [25]. Regarding the demographic profile, eight studies included exclusively male athletes, while five studies involved mixed-gender cohorts; no study evaluated female athletes exclusively. The aggregate sample size across all included studies was 176 participants, with individual sample sizes ranging from 8 to 27 athletes (Table 3). Participants were predominantly classified as amateurs (*n* = 8 studies), whereas four studies focused on elite-level competitors, and one study examined a mixed-status group. The mean age of the athletes generally ranged between 16 and 25 years, with the maximum age extending up to 29 years.

### 3.4. Meta-Analysis

Six studies evaluating the FSKT-10 and four studies assessing the TSAT yielded sufficient statistical parameters—specifically means and standard deviations—for inclusion. Given the paucity of comprehensive data for alternative performance metrics, FSKT-10 and TSAT were designated as the primary outcome measures. The quantitative synthesis of selected CAs extracted from the respective trials culminated in the incorporation of 34 CAs into the FSKT-10 meta-analytical model, and 21 CAs into the TSAT model (Table 4). Consequently, a subgroup meta-analysis based on combat sport disciplines was precluded due to limited data. The entire pool of extracted data for both performance indicators originated exclusively from studies involving taekwondo athletes.

#### 3.4.1. FSKT-10 Results

**Figure 2:**
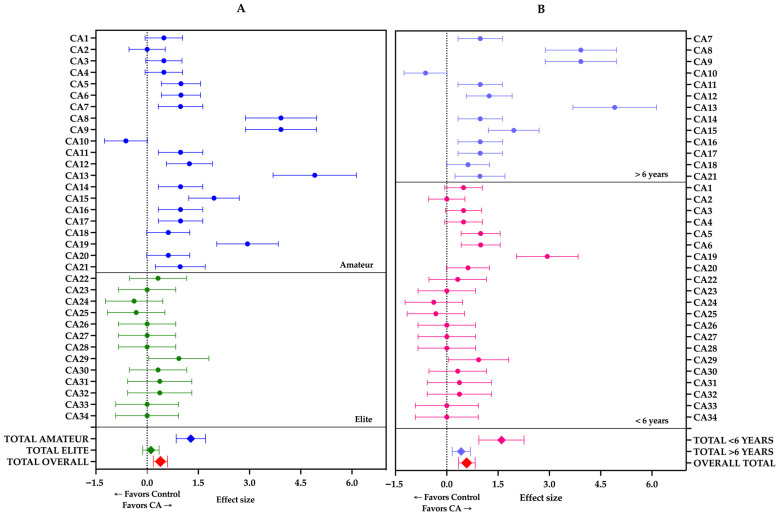
Forest plots (random-effects model) illustrating individual and aggregated Hedges’ effect sizes for the Frequency Speed of Kick Test (FSKT-10) performance, categorized by competitive level (**A**) and training experience (**B**). In panel (**A**), data points are color-coded for elite (green) and amateur (blue) athletes. In panel (**B**), data points represent athletes with <6 years (orchid) and >6 years (pink) of training experience. The overall pooled effect across all conditions is marked with a red diamond at the top of each plot. The pooled mean Hedges’ effect sizes were 0.39 (95% CI, 0.18 to 0.60) for competitive level and 0.58 (95% CI, 0.34 to 0.83) for training experience. Arrows below the horizontal axes indicate directionality, with data points falling to the left of the null line (0.0) favoring the control condition and those to the right favoring the Conditioning Activity (CA). The vertical dotted lines represent the overall pooled effect sizes, while the horizontal error bars indicate 95% confidence intervals.

The meta-analysis of the data relating to the effect of the competitive level factor (total *n* = 605) indicated significant differences between the groups (between-group Q = 21.60; *p* < 0.001). For amateur athletes (*n* = 470), the influence of potentiation led to a highly significant and large improvement in FSKT performance (g = 1.28; 95% CI, 0.85 to 1.71; *p* < 0.001) though considerable heterogeneity was observed within this subgroup (I^2^ = 88.71%). It was found that in several CAs, the mean for the ES was notably higher than the pooled mean, especially in the studies by Ouergui et al. [16] and Messaoudi et al. [20], which investigated dynamic, specific tasks. Conversely, elite athletes demonstrated a trivial and non-significant response to the CAs (g = 0.11; 95% CI, −0.13 to 0.35; *p* = 0.357) with a high degree of homogeneity across the included studies (I^2^ = 0%). In contrast to the amateur group, studies examining elite athletes—such as those by Da Silva Santos et al. [17,18] utilizing high-intensity resistance exercises like half squats and jumps—showed no substantial acute effect on FSKT-10 performance. Results are shown in Table 5 and Figure 2A.

Furthermore, the meta-analysis investigated the moderating effect of training experience, categorized into athletes with less than 6 years (<6; *n* = 268) and more than 6 years (>6; *n* = 337) of practice. A statistical analysis confirmed a significant difference between these two experience groups (between-group Q = 10.49; *p* = 0.001; I^2^ = 90.82%). Athletes with less than 6 years of practice demonstrated the most pronounced enhancements in FSKT-10 outcomes (g = 1.60; 95% CI, 0.94 to 2.26; *p* < 0.001; I^2^ = 62.88%). This large magnitude of improvement was predominantly driven by the interventions reported by Ouergui et al. [19]. On the other hand, athletes with more than 6 years of practice also exhibited a statistically significant improvement, albeit of a much smaller, moderate magnitude (g = 0.42; 95% CI, 0.16 to 0.69; *p* = 0.002). Within this more experienced cohort, which included the elite participants from Da Silva Santos et al. [17,18] as well as more experienced amateur groups from Ouergui et al. [19]. Results are shown in Table 5 and Figure 2B.

#### 3.4.2. TSAT Results

It should be noted that a subgroup meta-analysis based on competitive level was precluded for the TSAT, as the entirety of the extracted data for this metric originated exclusively from amateur athletes due to insufficient data. Consequently, the analysis focused solely on the moderating effect of training experience. Since the TSAT measures performance based on completion time, a negative effect size indicates a reduction in time, and thus, an improvement in performance.

**Figure 3:**
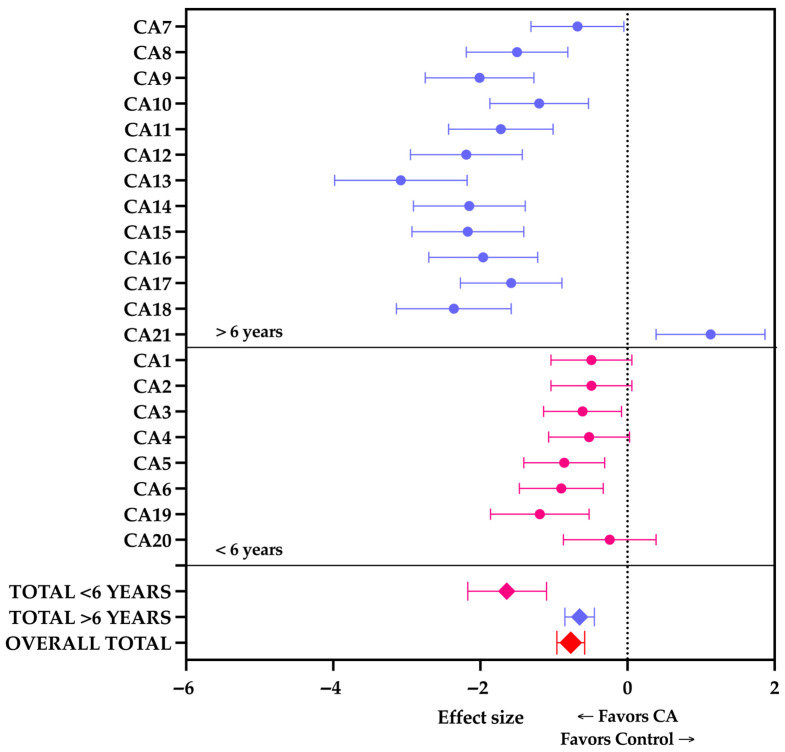
Forest plot (random-effects model) illustrating aggregated Hedges’ effect sizes for the Taekwondo-Specific Agility Test (TSAT) performance, categorized by training experience. The pooled mean Hedges’ effect size was −0.77 (95% CI, −0.96 to −0.58). Data points and their corresponding subgroup pooled effects (diamonds) are color-coded for athletes with <6 years (pink) and >6 years (purple) of training experience. The red diamond at the bottom represents the overall pooled effect across all conditions. The dotted vertical line represents the overall pooled effect size, while the error bars indicate 95% confidence intervals. As the TSAT is a timed metric, a negative effect size corresponds to a reduction in completion time; therefore, values to the left of the zero line indicate that the intervention favors CA. Arrows below the horizontal axes indicate directionality, with data points falling to the left of the null line (0.0) favoring the control condition and those to the right favoring the Conditioning Activity (CA).

The meta-analysis investigated the effect of training experience on TSAT performance, categorizing athletes into those with less than 6 years (<6; *n* = 268) and more than 6 years (>6; *n* = 202) of practice. The overall pooled results demonstrated a significant enhancement in agility following the conditioning activities (total *n* = 470; Hedges’ g = −0.77; 95% CI, −0.96 to −0.58; *p* < 0.001). Furthermore, the statistical analysis confirmed a significant difference in the magnitude of the PAPE response between the two experience groups (between-group Q = 11.44; *p* < 0.001). Athletes with less than 6 years of practice exhibited a large and highly significant improvement in TSAT times (Hedges’ g = −1.64; 95% CI, −2.17 to −1.10; *p* < 0.001), although considerable heterogeneity was observed within this subgroup (I^2^ = 85.68%). This pronounced effect was primarily driven by the specific dynamic interventions reported by Ouergui et al. [19]. Conversely, athletes with more than 6 years of practice also achieved a statistically significant enhancement in agility, though of a moderate magnitude (Hedges’ g = −0.65; 95% CI, −0.85 to −0.45; *p* < 0.001), with perfect homogeneity across the included studies (I^2^ = 0%). Results are shown in Table 6 and Figure 3.

### 3.5. Sensitivity Analysis

The results of the leave-one-study-out sensitivity analysis are presented in Table 7. For the FSKT-10 amateur subgroup (full model: g = 1.28; 95% CI: 0.85–1.71; I^2^ = 88.7%), sequential exclusion of individual studies revealed that Ouergui et al. [19] (12 conditioning activity arms) exerted the greatest influence on the pooled estimate. Its removal reduced I^2^ from 88.7% to 77.0%, while the pooled effect size decreased to g = 0.82 (95% CI: 0.41–1.24), remaining large and statistically significant. Exclusion of the remaining studies produced only marginal changes in both the effect size (range: g = 1.23–1.62) and heterogeneity (I^2^ = 88.5–90.6%), confirming that the high heterogeneity in this subgroup is structural, reflecting genuine variability in conditioning protocol design rather than the influence of a single outlying study.

For the TSAT subgroup comprising athletes with less than six years of training experience (full model: g = −1.64; 95% CI: −2.17–−1.10; I^2^ = 85.7%), Messaoudi et al. [20] was identified as the primary source of inconsistency. Exclusion of this study—which reported the only arm with a positive effect size (g = +1.13, indicating performance deterioration)—reduced I^2^ from 85.7% to 62.6%, while the pooled effect increased to g = −1.84 (95% CI: −2.19–−1.50), with all remaining arms showing consistent improvement in agility performance.

For the TSAT subgroup with more than six years of training experience, the full model demonstrated perfect homogeneity (I^2^ = 0.0%). Exclusion of Ouergui et al. [16] (six arms) left only two remaining arms, rendering the estimate unstable (g = −0.71; 95% CI: −1.64–0.22); however, this instability reflects insufficient data rather than a true reversal of effect. Exclusion of Ouergui et al. [21] (two arms) yielded g = −0.64 (95% CI: −0.87–−0.42; I^2^ = 0.0%), confirming the robustness of this subgroup’s estimate.

Across all subgroups and all iterations, the direction of the pooled effect was preserved, supporting the overall robustness of the primary conclusions.

To address the violation of the statistical independence assumption, a supplementary analysis was conducted in which effect sizes were aggregated to the study level prior to pooling. The results are presented in Table 8 alongside the original arm-level estimates.

For the FSKT-10 amateur subgroup, the study-level pooled effect size was g = 1.01 (95% CI: 0.56–1.45; k = 4 studies; *n* = 84 unique participants), compared to g = 1.28 in the arm-level analysis, remaining large and statistically significant. For the FSKT-10 elite subgroup, the study-level estimate was identical to the arm-level result (g = 0.11; 95% CI: −0.13–0.35; k = 2 studies; *n* = 20), confirming the absence of a significant effect. For the TSAT > 6 years subgroup, the study-level estimate likewise remained unchanged (g = −0.65; 95% CI: −0.85–−0.45; k = 2 studies; *n* = 47). In contrast, for the TSAT < 6 years subgroup, aggregation to study level yielded a non-significant pooled estimate (g = −0.35; 95% CI: −3.20–2.51; k = 2 studies; *n* = 37; I^2^ = 98.2%), reflecting the fundamental discrepancy between the two contributing studies.

### 3.6. Certainty of Evidence

The complete evidence profile is presented in Supplementary File S2. Overall, the certainty of evidence was rated as low to very low across all subgroups (Table 9).

For the FSKT-10 outcome, the certainty was rated as low for the overall pooled estimate and for the amateur and experience-based subgroups, primarily due to serious risk of bias (absence of blinding and inadequate allocation concealment), serious indirectness (outcomes derived exclusively from taekwondo athletes), and high heterogeneity in the amateur subgroup (I^2^ ≈ 88.7%). The certainty for the elite subgroup was downgraded to very low, additionally reflecting serious imprecision, as the confidence interval crossed zero and the effect was non-significant (g = 0.11; 95% CI: −0.13–0.35; *p* = 0.357).

For the TSAT outcome, the certainty was rated as low for the overall pooled estimate and the subgroup with more than six years of experience. The subgroup with less than six years of experience was rated as very low, due to very serious inconsistency (I^2^ = 85.68%) and very serious indirectness, as all data originated exclusively from young amateur taekwondo athletes studied within a single research group.

## 4. Discussion

The primary aim of this systematic review and meta-analysis, constituting Part II of our comprehensive evaluation in our recent work [4] was to quantitatively determine the PAPE on combat sport-specific performance outcomes, to examine whether athletic level and training experience moderate the magnitude of the PAPE response. The results indicate that PAPE interventions generally lead to significant improvements in sport-specific performance tasks in combat sports, particularly in FSKT-10 and TSAT. At the same time, the quantitative synthesis revealed a strong moderating effect of athletes’ training status. Specifically, amateur athletes and those with shorter training experience (<6 years) demonstrated large and highly significant improvements in sport-specific tests (Hedges’ g = 1.28 and 1.60 for the FSKT-10). Interpretation of these findings suggests that PAPE in combat sports is particularly pronounced in tasks requiring complex movement coordination and discipline-specific motor control. This interpretation is further supported by comparison with the results presented in Part I of our meta-analysis [4], which examined the influence of PAPE on general indicators of ballistic performance. In that analysis, the overall pooled effect size was relatively small (ES = 0.136) and did not differ significantly between amateur (ES = 0.14) and elite athletes (ES = 0.13). In contrast, the present meta-analysis, focusing on discipline-specific tasks, revealed clear differences between athletic levels. This may indicate that the moderating effect of training status becomes particularly evident in motor tasks characterized by a high degree of technical complexity, in which neuromuscular potentiation must interact with sport-specific motor control.

The notably large effect sizes reported in several subgroups (e.g., g = 1.28, 1.60, −1.64) warrant careful scrutiny and should be interpreted with caution for several reasons. First, as discussed above, the non-independence of arm-level observations may artificially inflate pooled estimates, a concern partially addressed by the study-level sensitivity analysis which yielded more conservative values (e.g., g = 1.01 for the FSKT-10 amateur subgroup). Second, the small per-study sample sizes (*n* = 9–27 participants) characteristic of the included studies are known to be associated with upward bias in effect size estimation, particularly under random-effects models. Third, the disproportionate contribution of a single research group (Ouergui et al. [16,19,21]), which accounts for the majority of conditioning activity arms in both outcomes, raises concerns regarding the generalizability of the pooled estimates beyond this specific laboratory context and participant population. Fourth, the absence of pre-registration for the majority of included studies increases the risk of selective outcome reporting, which may further contribute to effect size inflation. The supplementary study-level sensitivity analysis confirmed the primary findings for most subgroups; however, the TSAT < 6 years subgroup yielded a non-significant pooled estimate upon aggregation (g = −0.35; 95% CI: −3.20–2.51), highlighting the fragility of this particular finding. Future meta-analyses should consider multilevel meta-analytic models to formally accommodate the nested structure of multi-arm study designs.

In the present study, the limited PAPE response observed among elite athletes may be associated with their high baseline neuromuscular performance and proximity to a functional performance “ceiling effect”. Highly trained athletes typically exhibit well-developed neuromuscular characteristics, including efficient motor unit recruitment and enhanced neural drive, which may result from long-term training adaptations and may limit the potential for further acute improvements [29]. Similar differences between athletic levels were reported by James et al. [30], who found that higher-level mixed martial arts athletes demonstrated greater lower-limb maximal strength, higher rate of force development, and superior jump performance compared with lower-level competitors. Although that study did not directly examine PAPE, it supports the notion that elite athletes possess more developed neuromotor characteristics, which may partly explain the smaller magnitude of additional potentiation observed in the present meta-analysis. At the same time, the findings of the present study differ from conclusions reported in some previous meta-analyses examining PAP/PAPE phenomena. Seitz and Haff [31] suggested that stronger individuals may exhibit greater PAP effects (ES = 0.41) compared with weaker individuals (ES = 0.32). Similar tendencies were reported in the meta-analysis by Xu et al. [32], in which highly trained athletes (ES = 0.38) responded more strongly to CA than recreationally trained individuals (ES = 0.21) and physically active participants (ES = 0.22). Furthermore, participants with at least two years of training experience (ES = 0.36) demonstrated greater responses than those with shorter training experience (ES = 0.16). However, such a relationship was not observed in the present meta-analysis. One possible explanation for this discrepancy may lie in the specific characteristics of the analyzed population of combat sport athletes. To our knowledge, this is the first study to directly examine training status as a moderator of PAPE in combat sports. It should be noted that in the included studies, competitive level and training experience were substantially overlapping classifications, a limitation discussed in greater detail below. We hypothesize that such a substantial difference in training exposure corresponds to distinct levels of neuromotor adaptation. While direct empirical evidence in combat sports is scarce, foundational physiological principles suggest that chronic neural adaptations in elite athletes may attenuate the relative magnitude of acute performance enhancement [29,33]. Furthermore, as demonstrated in mixed martial arts competitors, advanced athletes exhibit superior resistance to neuromuscular fatigue, maintaining lower limb reaction times under significantly higher external loads compared to less experienced fighters [34,35,36]. This enhanced capacity to tolerate heavy resistance may alter the individual potentiation-to-fatigue ratio, partially explaining the blunted PAPE responsiveness observed in highly trained cohorts. In contrast to elite athletes, amateur and less experienced athletes demonstrated very large effects in our analysis. This may reflect greater neuromuscular plasticity in individuals with lower training status, who typically exhibit larger relative adaptations to training stimuli compared with well-trained populations [37,38]. As derived from the large effect sizes observed in our analysis, lower baseline levels of strength and less stabilized coordination patterns may allow transient increases in central neural drive to translate more effectively into kinetic improvements during complex motor tasks. This aligns with findings suggesting that less experienced individuals exhibit greater acute neuromuscular plasticity in response to conditioning stimuli compared to their highly trained counterparts [38,39]. These findings suggest a greater adaptive reserve in less trained athletes and indicate that athletic level may represent an important moderator of potentiation in combat sports.

The results of this meta-analysis also highlight the important role of the type of CAs used. The largest performance improvements were observed in studies employing dynamic and biomechanically compatible protocols, such as repeated high-velocity kicking sequences or plyometric exercises. This may suggest that the effectiveness of PAPE in combat sports largely depends on the neuromotor specificity between the CA and the target task. In these disciplines, technical effectiveness requires rapid force generation under conditions of complex intersegmental coordination [40,41]. Therefore, stimulus specificity may play a crucial role in the transfer of potentiation [42]. In several studies [18,26], traditional high-intensity resistance protocols, such as heavy half squats, did not lead to substantial improvements in technical performance. This may be explained by limited task specificity or insufficient individualization of load parameters and rest intervals. These observations are consistent with previous meta-analyses investigating the type of CA. Seitz and Haff [31] reported that plyometric exercises may be slightly more effective than traditional resistance exercises in eliciting potentiation. Similar conclusions were presented in the meta-analysis by Xu et al. [32], in which plyometric exercises (ES = 0.42) produced larger effects than traditional resistance exercises (ES = 0.23), maximal voluntary isometric contractions (ES = 0.31), and other forms of CAs (ES = 0.24). These observations are further supported by the recent narrative review by Franchini and Takito [3], which emphasized that PAPE responses in combat sports are shaped by the interaction between the type of CA, the recovery interval, and the specificity of the target task. In taekwondo, acute improvements have been reported after both traditional strength–power protocols and sport-specific kicking-based conditioning activities. However, the magnitude of the response appears to depend on the recovery interval and the performance outcome, with plyometric stimuli generally favored after ~3 min and repeated high-intensity kicking sequences benefiting more from longer intervals of ~7–10 min in repeated-kick and agility tasks [16,17,19]. Similar patterns have been observed in judo, where plyometric and contrast-type activities, including standing broad jumps and resistance-band pulls, enhanced SJFT performance, particularly in the first set, despite relatively short recovery periods of 1–7 min [24,27,43]. However, when a different judo-specific outcome was assessed, a dedicated sport-specific warm-up was superior to more general routines, suggesting that the transfer of PAPE depends not only on stimulus intensity, but also on the biomechanical and coordinative correspondence between the CA and the subsequent task [44].

The supplementary GRADE analysis indicated that the certainty of evidence for both FSKT-10 and TSAT outcomes was rated as low to very low, primarily due to imprecision (small sample sizes), inconsistency (high I^2^ values in several subgroups), and indirectness (homogeneous taekwondo samples limiting generalizability). The sensitivity analysis confirmed that the substantial heterogeneity observed in the FSKT-10 amateur subgroup is structural in nature, as no single study removal reduced I^2^ below 77%, and the large effect remained significant in all iterations. In the TSAT < 6 years subgroup, Messaoudi et al. [20] was identified as the primary source of inconsistency. However, its exclusion further strengthened the pooled estimate, suggesting that the true effect in this subgroup may be even larger than reported.

A critical gap identified by the present systematic search concerns the complete absence of studies exclusively investigating female combat sports athletes. Of the 13 included studies, five involved mixed-gender cohorts, while the remaining eight comprised exclusively male participants. No study was identified that examined PAPE effects in a purely female sample. This underrepresentation likely reflects broader structural issues in sports science research, including the historical predominance of male participants in exercise physiology and combat sports research, smaller competitive female athlete pools in some disciplines, and potential publication bias favoring studies with larger, predominantly male samples. Given established sex-based differences in muscle fiber type composition, hormonal milieu, neuromuscular fatigue resistance, and baseline strength levels—all of which may influence the potentiation-to-fatigue ratio underlying PAPE—the extrapolation of the present findings to female combat sports athletes is not warranted.

An important additional conceptual consideration concerns the interpretation of improvements observed in sport-specific tests such as the FSKT-10 and TSAT. Unlike simple ballistic tasks (e.g., countermovement jump or sprint), these assessments involve complex motor demands, including intersegmental coordination, movement timing, technical execution, and pacing strategy. Consequently, performance variations in these tests cannot be unequivocally attributed to PAPE mechanisms alone. Several alternative or complementary explanations warrant acknowledgement. First, familiarization effects may inflate performance estimates in crossover designs, particularly in studies with limited wash-out periods or insufficient practice trials prior to testing. Second, pacing adjustments—whereby athletes consciously or unconsciously modulate effort distribution across repeated kicking or agility sequences following a CA—may independently contribute to observed improvements. Third, acute changes in arousal, motivation, or attentional focus induced by the CA itself may transiently enhance technical performance through psychophysiological pathways unrelated to myosin light chain phosphorylation or post-tetanic potentiation. Fourth, the warm-up effect per se, rather than the specific potentiating stimulus, may account for a portion of the observed gains, particularly in studies where the CA was not adequately distinguished from general warm-up procedures.

Nevertheless, other limitations of this meta-analysis should be acknowledged. Firstly, the quantitative synthesis for specific performance indicators (FSKT and TSAT) was entirely derived from taekwondo athletes. Consequently, the generalizability of these meta-analytic findings to grappling disciplines (e.g., wrestling, Brazilian jiu-jitsu), which heavily rely on sustained isometric contractions and different bioenergetic pathways, remains constrained. Furthermore, considerable heterogeneity existed among studies in terms of CA protocols, rest interval durations, and testing procedures. This limitation is particularly important from an applied perspective. Experimental studies most commonly examine single-exercise protocols, whereas practitioners often implement multi-exercise formats, which limits the ecological validity and direct transferability of the current evidence [45]. A further interpretive limitation concerns the potential collinearity between the two moderating variables examined in the present meta-analysis—competitive level (amateur vs. elite) and training experience (<6 vs. >6 years). Inspection of the included studies reveals substantial overlap between these classifications: elite athletes were consistently characterized by training histories exceeding six years, while amateur athletes predominantly fell within the <6 years category. Consequently, these moderators should not be interpreted as fully independent, and the observed between-subgroup differences likely reflect a composite of competitive level, accumulated training experience, neuromuscular adaptation status, and habitual training load rather than the isolated influence of either variable alone. Whereas the FSKT-10 analysis permitted subgroup comparisons across both competitive level (amateur vs. elite) and training experience (<6 vs. >6 years), the TSAT analysis was restricted to the training experience moderator only, as all available TSAT data originated exclusively from amateur athlete cohorts. The absence of TSAT data from elite athletes precludes direct comparison of competitive level effects across both outcomes and limits the generalizability of the experience-based TSAT findings. It cannot be excluded that elite athletes would demonstrate a different pattern of TSAT responses to PAPE interventions than that observed in the present amateur sample.

### Practical Applications

The findings of this meta-analysis offer several practical implications for coaches and sport scientists working with combat sports athletes. First, amateur athletes and those with less than six years of training experience represent the primary beneficiary group for sport-specific PAPE protocols and should be prioritized accordingly. Second, dynamic, biomechanically specific conditioning activities—such as repeated high-intensity kicking sequences and plyometric exercises—appear more effective than traditional resistance protocols for potentiating FSKT-10 and TSAT performance and should be preferred in pre-competition priming routines. Third, rest intervals of approximately 7–10 min following high-intensity kicking sequences and approximately 3 min following plyometric stimuli may optimize the potentiation-to-fatigue ratio, though individual adjustment is recommended. Finally, coaches working with elite or highly experienced athletes should be aware that standard PAPE protocols may yield attenuated responses in this population, necessitating more individualized priming strategies.

The present findings reveal that the PAPE literature in combat sports remains in its early stages, and several fundamental questions are yet to be answered. Future research should prioritize the development of validated sport-specific outcome measures across grappling disciplines to enable broader cross-disciplinary synthesis, move beyond categorical athlete classifications toward continuous indicators of neuromuscular adaptation status to better explain who benefits from PAPE and why, and—most urgently—address the complete absence of female athletes from the quantitative evidence base, as no practice recommendations can be responsibly extended to this population without dedicated empirical investigation.

## Figures and Tables

**Table 1:** PICOS framework.

Factor	Description
P	Healthy combat sports athletes actively participating in recognized disciplines such as boxing, judo, karate, taekwondo, Brazilian jiu-jitsu, MMA, wrestling, kickboxing, and Muay Thai. Athletes must be free from injury and any health condition that could affect neuromuscular or sport-specific performance.
I	Studies must implement a clearly defined CA, specifying training parameters such as intensity (e.g., %1RM or equivalent load prescription), volume (sets/reps), intra- and interset rest intervals, and exercise type. Sport-specific performance must be assessed pre- and post-intervention.
C	Control or comparator conditions, including passive rest, standard warm-up, alternative activation strategies, or between different PAPE protocols. Studies may also compare responses across disciplines, competitive level, or athlete training experience.
O	Acute changes in sport-specific performance assessed using validated combat-sport tests, (e.g., total kicks/score, time-based indices, decrement/fatigue index), sport-specific striking/kicking combinations, or discipline-specific task simulations. Results must be reported with means and standard deviations, change scores, or effect sizes sufficient to compute standardized effects.
S	Experimental designs such as randomized controlled trials, randomized or non-randomized crossover designs, or quasi-experimental studies with clearly defined protocols and sport-specific outcome measures.

P—participants; I—interventions; C—comparisons; O—outcomes; S—study design.

**Table 2:** PEDro scale quality assessment.

#	Reference	1	2	3	4	5	6	7	8	9	10	11	Sum
1	Ouergui et al., 2022a [16]	yes	1	1	1	1	0	1	1	1	1	1	9
2	Da Silva Santos et al., 2015 [17]	yes	1	1	1	1	0	1	1	1	1	1	9
3	da Silva Santos et al., 2016 [18]	yes	1	1	1	1	0	1	1	1	1	1	9
4	Ouergui et al., 2023 [19]	yes	1	1	0	1	1	1	1	1	1	1	9
5	Messaoudi et al., 2024 [20]	yes	1	0	1	0	0	0	1	1	1	1	6
6	Ouergui et al., 2022b [21]	yes	1	0	1	0	0	0	1	1	1	1	6
7	Finlay et al., 2022 [22]	yes	1	1	1	1	0	1	1	1	1	1	9
8	Yi et al., 2022 [23]	yes	1	0	1	0	0	1	1	1	1	1	7
9	Miarka et al., 2011 [24]	yes	1	0	1	0	0	0	1	1	1	1	6
10	Margaritopoulos et al., 2015 [25]	yes	1	1	1	1	0	1	1	1	1	1	9
11	Castro-Garrido et al., 2020 [26]	yes	1	1	1	1	0	1	1	1	1	0	8
12	Lum, 2019 [27]	yes	1	1	1	1	0	1	1	1	0	1	8
13	Oliveira et al., 2018 [28]	yes	1	0	1	1	0	1	1	1	0	1	7

**Table 3:** Characteristics of studies included in systematic review.

Ref.	AGE (M ± SD)	SEX	Level	1RM	D	n	CG	CA	L	RI, min	ISR, s	SPT	Main Outcomes
Yi et al., 2022 [23]	19.20 ± 1.55	M	A	90.80 ± 8.39 kg SQ	BOX	10	N	LSJ SQ	LSJ: 4 × 8@30% SQ: 3 × 5@80%	3/6/9/12	90	RHSP	↑ force & speed
Miarka et al., 2011 [24]	19.0 ± 1.0	M	A	X	JU	8	Y	VJ SQ SQ + HJ	VJ: 10 × 3 SQ: 5 × 1@95% SQ + HJ: 3 × 2@90% + 5	3	30/120	SJFT	↑
Ouergui et al., 2022a [16]	16.0 ± 1.0	MIX	A	X	TKD	27	Y	RHIT P	RHIT: 3 × 5 s P: 3 × 5 s	10	Ratio/SSR	TSAT FSKT-10s FSKT-mult	↑
Margaritopoulos et al., 2015 [25]	18.4 ± 1.2 (M) 19.2 ± 0.4 (F)	MIX	E	X	KAR	10	Y	TJ	3 × 5	<1	30	RKF	↑
Messaoudi et al., 2024 [20]	19.94 ± 1.12	M	A	X	TKD	16	Y	P	P: 3 × 5 s (40 cm)	3	NR	TSAT, FSKT-10s, FSKT-mult	↑
Da Silva Santos et al., 2015 [17]	20.3 ± 5.2	M	E	136.4 ± 30.7 kg HS	TKD	11	Y	HS J HS + J	HS: 3 × 1@95% J: 3 × 10 (40 cm) HS + J: 3 × 2@95% + 4	5/10/SSR	30/180	FSKT-10s	↑
Castro-Garrido et al., 2020 [26]	20.50 ± 2.38 (A) 24.75 ± 4.27 (E)	M	MIX	X	TKD	8	Y	HS HS + J J	HS: 3 × 3@95% HS + J: 3 × 2@95% + 4 J: 3 × 10	10	30/180	FSKT-10s FSKT-mult	↔
da Silva Santos et al., 2016 [18]	20.3 ± 5.2	M	E	132.8 ± 32.5 kg HS	TKD	9	Y	HS	1 × 3 (50%) 1 × 3 (90%) 3 × 3 (50%) 3 × 3 (90%)	10	NR	FSKT-10s	↔
Lum et al., 2019 [27]	16–29	M	E	X	JU	11	Y	ERP + BJ BJ	ERP + BJ: 2 × 5 or 3 × 5 BJ: 3 × 5	5	60	SJFT	↑
Oliveira et al., 2018 [28]	18.6 ± 2.1	MIX	A	X	TKD	15	N	WBV	1 min@26 Hz	NR	NR	RHK	↔
Ouergui et al., 2022b [21]	17.5 ± 0.7	MIX	A	X	TKD	20	Y	P	P: 3 × 10 (40 cm)	10	NR	TSAT FSKT-10s FSKT-mult	↑
Ouergui et al., 2023 [19]	20.4 ± 1.4	MIX	A	X	TKD	21	Y	RHIT P	RHIT: 3 × 5 s P: 3 × 5 s (40 cm)	3/7	1:6/1:9/SSR	TSAT FSKT-10s FSKT-mult	↑
Finlay et al., 2022 [22]	19.7 ± 1.2	M	A	X	BOX	10	Y	ELRP ISOP	ELRP: 2 × 5 ISOP: 3 × 3s	3/5/7/9/11/13	NR	Punches (Jab, cross, hooks)	↑/↔/↓

1RM—one maximal repetition test; A—amateur; BJ—broad jump; BOX—boxing; CA—conditioning activity; CG—control group; DJ—drop jump; E—elite; ERP—elastic resistance pull; ELRP—elastic resistance punch; ISOP—isometric punch; F—female; FSKT-10s—10 s frequency speed of kick test; FSKT-mult—multiple frequency speed of kick test; HJ—horizontal jump; HS—half squat; ISR—intra-set rest; J—jump; JU—judo; KAR—karate; L—load as SETS × REPS@INTENSITY; LSJ—loaded squat jump; M—mean; M—male; MIX—mixed (for sex: males and females; for discipline: MMA, Muay Thai, Kickboxing); n—sample size; N—no; NR—not reported; P—plyometrics; RHIT—repeated high-intensity techniques; RHK—roundhouse kick; RHSP—rear-hand straight punch; RI—rest intervals; RKF—round kick force; SD—standard deviation; SJFT—Special Judo Fitness Test; SPT—specific performance test; SQ—squat; SSR—self-selected rest interval; TJ—tuck jump; TKD—taekwondo; TSAT—taekwondo-specific agility test; VJ—vertical jump; WBV—whole-body vibration; X—lack of data; Y—yes; ↑ significant improvement; ↔ no significant change; ↓ significant deterioration.

**Table 4:** Conditioning activities of studies included in the meta-analysis.

Author	#	Conditioning Activity/Competitive Level/Experience/Test
Ouergui et al., 2022a [16]	CA1	Bandal chagui, 3 × 5 s, 10 min rest, 30 s intraset rest/A/>6/FSKT-10 + TSAT
	CA2	Bandal chagui, 3 × 5 s, 10 min rest, 35 s intraset rest/A/>6/FSKT-10 + TSAT
	CA3	Bandal chagui, 3 × 5 s, 10 min rest, self-selected intraset rest/A/>6/FSKT-10 + TSAT
	CA4	Consecutive vertical jump, 3 × 5 s, 10 min rest, 30 s intraset rest/A/>6/FSKT-10 + TSAT
	CA5	Consecutive vertical jump, 3 × 5 s, 10 min rest, 35 s intraset rest/A/>6/FSKT-10 + TSAT
	CA6	Consecutive vertical jump, 3 × 5 s, 10 min rest, self-selected interset rest/A/>6/FSKT-10 + TSAT
Ouergui et al., 2023 [19]	CA7	Bandal chagui, 3 × 5 s, 3 min rest, 30 s intraset rest/A/<6/FSKT-10 + TSAT
	CA8	Bandal chagui, 3 × 5 s, 3 min rest, 45 s intraset rest/A/<6/FSKT-10 + TSAT
	CA9	Bandal chagui, 3 × 5 s, 3 min rest, self-selected intraset rest/A/<6/FSKT-10 + TSAT
	CA10	Consecutive vertical jump, 3 × 5 s, 3 min rest, 30 s intraset rest/A/<6/FSKT-10 + TSAT
	CA11	Consecutive vertical jump, 3 × 5 s, 3 min rest, 45 s intraset rest/A/<6/FSKT-10 + TSAT
	CA12	Consecutive vertical jump, 3 × 5 s, 3 min rest, self-selected interset rest/A-<6/FSKT-10 + TSAT
	CA13	Bandal chagui, 3 × 5 s, 7 min rest, 30 s intraset rest/A/<6/FSKT-10 + TSAT
	CA14	Bandal chagui, 3 × 5 s, 7 min rest, 45 s intraset rest/A/<6/FSKT-10 + TSAT
	CA15	Bandal chagui, 3 × 5 s, 7 min rest, self-selected intraset rest/A/<6/FSKT-10 + TSAT
	CA16	Consecutive vertical jump, 3 × 5 s, 7 min rest, 30 s intraset rest/A/<6/FSKT-10 + TSAT
	CA17	Consecutive vertical jump, 3 × 5 s, 7 min rest, 45 s intraset rest/A/<6/FSKT-10 + TSAT
	CA18	Consecutive vertical jump, 3 × 5 s, 7 min rest, self-selected interset rest/A/<6/FSKT-10 + TSAT
Ouergui et al., 2022b [21]	CA19	Vertical jumps 3 × 10, 10 min rest/A/>6/FSKT-10 + TSAT
	CA20	Control, 10 min rest/A/>6/FSKT-10 + TSAT
Messaoudi et al., 2024 [20]	CA21	Consecutive vertical jump 3 × 5 s, 3 min rest/A/<6/FSKT-10 + TSAT
Da Silva Santos et al., 2015 [17]	CA22	Half squat, 3 × 1 × 95%1RM, 5 min rest, 3 min interset rest/E/>6/FSKT-10
	CA23	Half squat, 3 × 1 × 95%1RM, 10 min rest, 3 min interset rest/E/>6/FSKT-10
	CA24	Half squat, 3 × 1 × 95%1RM, self-selected rest, 3 min interset rest/E/>6/FSKT-10
	CA25	Jumps, 3 × 10, 5 min rest, 30 s interset rest/E/>6/FSKT-10
	CA26	Jumps, 3 × 10, 10 min rest, 30 s interset rest/E/>6/FSKT-10
	CA27	Jumps, 3 × 10, self-selected rest, 30 s interset rest/E/>6/FSKT-10
	CA28	Half squat + Jumps, 3 × 2 × 95%1RM + 4, 5 min rest, 3 min interset rest/E/>6/FSKT-10
	CA29	Half squat + Jumps, 3 × 2 × 95%1RM + 4, 10 min rest, 3 min interset rest/E/>6/FSKT-10
	CA30	Half squat + Jumps, 3 × 2 × 95%1RM + 4, self-selected rest, 3 min interset rest/E/>6/FSKT-10
Da Silva Santos et al., 2016 [18]	CA31	Half squat, 1 × 3 × 50%1RM, 10 min rest/E/>6/FSKT-10
	CA32	Half squat, 1 × 3 × 90%1RM, 10 min rest/E/>6/FSKT-10
	CA33	Half squat, 3 × 3 × 50%1RM, 10 min rest/E/>6/FSKT-10
	CA34	Half squat, 3 × 3 × 90%1RM, 10 min rest/E/>6/FSKT-10

1RM—one maximal repetition test; competition level: A—amateur, E—elite; CA—conditioning activity.

**Table 5:** Overview of the FSKT-10 meta-analysis by competitive level and years of practice—aggregated view.

Subgroup	Sample Size	ES	SE	±95% CI	Z-Statistic	*p*-Value	Variance	Weight	Contribution
Competitive Level									
Amateur	470	1.28	0.22	0.85; 1.71	5.85	<0.001 *	0.05	20.83	0.24
Elite	135	0.11	0.12	−0.13; 0.35	0.92	0.357	0.02	65.94	0.76
*Total*	*605*	*0.39*	*0.11*	*0.18*; *0.60*	*3.67*	<*0.001* *			
Experience									
<6 years	268	1.60	0.34	0.94; 2.26	4.74	<0.001 *	0.11	8.78	0.14
>6 years	337	0.42	0.13	0.16; 0.69	3.13	0.002 *	0.02	55.26	0.86
*Total*	*605*	*0.58*	*0.12*	*0.34*; *0.83*	*4.67*	<*0.001* *			

SE—standard error; ±95% CI—confidence interval (lower; upper); *—statistical significance; ES—effect size. Italics denote the overall pooled results.

**Table 6:** Overview of the TSKT meta-analysis by competitive level and years of practice—aggregated view.

Subgroup	Sample Size	ES	SE	±95% CI	Z-Statistic	*p*-Value	Variance	Weight	Contribution
<6 years	268	−1.64	0.27	−2.17; −1.10	−5.99	<0.001 *	0.07	13.38	0.12
>6 years	202	−0.65	0.10	−0.85; −0.45	−6.34	<0.001 *	0.01	95.04	0.88
*Total*	*470*	−*0.77*	*0.10*	−*0.96*; −*0.58*	−*8.04*	<*0.001* *			

SE—standard error; ±95% CI—confidence interval (lower; upper); *—statistical significance; ES—effect size. Italics denote the overall pooled results.

**Table 7:** Leave-one-study-out sensitivity analysis for primary outcomes.

Outcome	Study Removed	Arms Removed	Remaining Arms	g	95% CI	I^2^
FSKT-10 Amateur	Full model	—	21	1.28	0.85–1.71	88.7%
	Ouergui et al. [16]	6	15	1.62	1.01–2.22	90.6%
	Ouergui et al. [19]	12	9	0.82	0.41–1.24	77.0%
	Ouergui et al. [21]	2	19	1.23	0.79–1.68	88.5%
	Messaoudi et al. [20]	1	20	1.30	0.85–1.75	89.3%
TSAT < 6 years	Full model	—	13	−1.64	−2.17–−1.10	85.7%
	Messaoudi et al. [20]	1	12	−1.84	−2.19–−1.50	62.6%
TSAT > 6 years	Full model	—	8	−0.65	−0.85–−0.45	0.0%
	Ouergui et al. [16]	6	2	−0.71	−1.64–0.22	75.8% *
	Ouergui et al. [21]	2	6	−0.64	−0.87–−0.42	0.0%

* Instability reflects insufficient remaining arms (k = 2) rather than a true reversal of effect.

**Table 8:** Study-level aggregation.

Subgroup	Arm-Level			Study-Level		
	k (Arms)/n	g	95% CI	k (Studies)/n	g	95% CI
FSKT-10 Amateur	21/470	1.28	0.85–1.71	4/84	1.01	0.56–1.45
FSKT-10 Elite	13/135	0.11	−0.13–0.35	2/20	0.11	−0.13–0.35
TSAT < 6 years	13/268	−1.64	−2.17–−1.10	2/37	−0.35 *	−3.20–2.51
TSAT > 6 years	8/202	−0.65	−0.85–−0.45	2/47	−0.65	−0.85–−0.45

* Non-significant; 95% CI crosses zero. k = number of arms or studies; n = total observations (arm-level) or unique participants (study-level).

**Table 9:** Summary of GRADE certainty ratings for primary outcomes.

Outcome	Subgroup	Hedges’ g	Certainty
FSKT-10	Overall	0.39	Low
FSKT-10	Amateur	1.28	Low
FSKT-10	Elite	0.11	Very Low
FSKT-10	<6 years	1.60	Low
FSKT-10	>6 years	0.42	Low
TSAT	Overall	−0.77	Low
TSAT	<6 years	−1.64	Very Low
TSAT	>6 years	−0.65	Low

Full GRADE evidence profile including domain-level ratings is presented in Supplementary File S2.

## Data Availability

The raw data supporting the conclusions of this article will be made available by the authors on request.

## Supplementary Materials

The following supporting information can be downloaded at: https://www.mdpi.com/article/10.3390/jfmk11020157/s1, File S1: PRISMA checklist; File S2: GRADE Evidence Profile.

## Abbreviations

The following abbreviations are used in this manuscript:

1RM	One maximal repetition test
A	Amateur
BJ	Broad jump
BJJ	Brazilian jiu-jitsu
BOX	Boxing
CA	Conditioning activity
CG	Control group
CI	Confidence interval
D	Discipline
DJ	Drop jump
E	Elite
ELRP	Elastic resistance punch
ERP	Elastic resistance pull
ES	Effect size
F	Female
FSKT/FSKT-10s	Frequency Speed of Kick Test (10 s)
FSKT-mult	Multiple frequency speed of kick test
GRADE	Grading of Recommendations Assessment, Development and Evaluation
HJ	Horizontal jump
HS	Half squat
ISOP	Isometric punch
ISR	Intra-set rest
J	Jump
JU	Judo
KAR	Karate
L	Load as SETS × REPS@INTENSITY
LSJ	Loaded squat jump
M	Mean/Male
MIX	Mixed (for sex: males and females; for discipline: MMA, Muay Thai, Kickboxing)
MMA	Mixed martial arts
n	Sample size
N	No
NR	Not reported
P	Plyometrics/Participants
PAP	Post-activation potentiation
PAPE	Post-activation performance enhancement
PEDro	Physiotherapy Evidence Database
PICOS	Participants, Interventions, Comparisons, Outcomes, Study design
PRISMA	Preferred Reporting Items for Systematic Reviews and Meta-Analyses
RHIT	Repeated high-intensity techniques
RHK	Roundhouse kick
RHSP	Rear-hand straight punch
RI	Rest intervals
SD	Standard deviation
SE	Standard error
SJFT	Special Judo Fitness Test
SPT	Specific performance test
SQ	Squat
SSR	Self-selected rest interval
TJ	Tuck jump
TKD	Taekwondo
TSAT	Taekwondo-specific agility test
VJ	Vertical jump
WBV	Whole-body vibration
X	Lack of data
Y	Yes
