# Supplementary material for: Effect of Post-Activation Performance Enhancement in Combat Sports: A Systematic Review and Meta-Analysis-Part II: Specific Performance Indicators
Source: J Funct Morphol Kinesiol. 2026 Apr 16;11(2):157. doi: 10.3390/jfmk11020157 (PMC13108071; doi:10.3390/jfmk11020157)
Supplement: Supplementary file 1 [file jfmk-11-00157-s001.zip › jfmk-4215414-File S2-GRADE Evidence Profile.pdf]

# Supplementary file S2 — GRADE Evidence Profile

| GRADE Evidence Profile — PAPE Effects on Sport-Specific Performance in Combat Sports | | | | | | | | |
|---|---|---|---|---|---|---|---|---|
| **Outcome** | **No. of Studies (n)** | **Risk of Bias** | **Inconsistency** | **Indirectness** | **Imprecision** | **Publication Bias** | **Effect Size (Hedges' g)** | **Certainty of Evidence** |
| ***Frequency Speed of Kick Test — 10 s (FSKT-10)*** | | | | | | | | |
| FSKT-10 overall (all athletes) | 8 studies (n = 605) | Serious ↓ (no blinding; non-randomized crossovers) | Serious ↓ ($I^2$ = 73–91%; high heterogeneity) | Serious ↓ (taekwondo only; limited sport diversity) | Serious ↓ (small per-study n; wide CIs in subgroups) | Not detected (insufficient n for funnel plot) | **g = 0.39 (95% CI: 0.18–0.60)** | **⊕⊕○○ LOW** |
| FSKT-10 — Amateur athletes | 5 studies (n = 403) | Serious ↓ (no blinding; allocation not always concealed) | Very serious ↓↓ ($I^2$ = 80–91%) | Serious ↓ (taekwondo only) | Not serious (consistent direction; CIs exclude 0) | Not detected | **g = 1.28 (95% CI: 0.72–1.84) p < 0.001** | **⊕⊕○○ LOW** |
| FSKT-10 — Elite athletes | 3 studies (n = 202) | Serious ↓ (no blinding) | Not serious ($I^2$ = 0–28%) | Serious ↓ (taekwondo only) | Very serious ↓↓ (n < 50 total; wide CIs; effect crosses 0) | Not detected | **g = 0.11 (95% CI: −0.13–0.35) p = 0.357** | **⊕○○○ VERY LOW** |
| FSKT-10 — <6 years experience | 4 studies (n = 268) | Serious ↓ (no blinding) | Serious ↓ ($I^2$ = 62.88%) | Serious ↓ (taekwondo only) | Not serious (consistent large effect; CIs exclude 0) | Not detected | **g = 1.60 (95% CI: 0.94–2.26) p < 0.001** | **⊕⊕○○ LOW** |
| FSKT-10 — >6 years experience | 4 studies (n = 337) | Serious ↓ (no blinding) | Not serious (moderate $I^2$) | Serious ↓ (taekwondo only) | Not serious (CIs exclude 0) | Not detected | **g = 0.42 (95% CI: 0.16–0.69) p = 0.002** | **⊕⊕○○ LOW** |
| ***Taekwondo Specific Agility Test (TSAT) — time in seconds (negative g = improvement)*** | | | | | | | | |
| TSAT overall (all athletes) | 5 studies (n = 470) | Serious ↓ (no blinding; crossover designs) | Serious ↓ ($I^2$ = 85.68% in <6yr subgroup) | Very serious ↓↓ (taekwondo only; amateurs only for TSAT) | Not serious (large pooled n; narrow overall CI) | Not detected | **g = −0.77 (95% CI: −0.96–−0.58) p < 0.001** | **⊕⊕○○ LOW** |
| TSAT — <6 years experience | 3 studies (n = 268) | Serious ↓ (no blinding) | Very serious ↓↓ ($I^2$ = 85.68%) | Very serious ↓↓ (taekwondo amateurs only) | Not serious (large effect; CIs exclude 0) | Not detected | **g = −1.64 (95% CI: −2.17–−1.10) p < 0.001** | **⊕○○○ VERY LOW** |
| TSAT — >6 years experience | 2 studies (n = 202) | Serious ↓ (no blinding) | Not serious ($I^2$ = 0%) | Very serious ↓↓ (taekwondo amateurs only) | Not serious (CIs exclude 0) | Not detected | **g = −0.65 (95% CI: −0.85–−0.45) p < 0.001** | **⊕⊕○○ LOW** |

**GRADE certainty ratings: ⊕⊕⊕⊕ High | ⊕⊕⊕○ Moderate | ⊕⊕○○ Low | ⊕○○○ Very Low**

Reasons for downgrading: Risk of bias — lack of blinding and non-randomized designs; Inconsistency — high $I^2$ statistic; Indirectness — outcomes derived exclusively from taekwondo athletes, limiting generalizability to other combat sports; Imprecision — small sample sizes and wide confidence intervals in subgroups. ↓ = one level downgrade; ↓↓ = two level downgrade.
